# Glypican-1 is enriched in circulating-exosomes in pancreatic cancer and correlates with tumor burden

**DOI:** 10.18632/oncotarget.24873

**Published:** 2018-04-10

**Authors:** Adam E. Frampton, Mireia Mato Prado, Elena López-Jiménez, Ana Belen Fajardo-Puerta, Zaynab A.R. Jawad, Phillip Lawton, Elisa Giovannetti, Nagy A. Habib, Leandro Castellano, Justin Stebbing, Jonathan Krell, Long R. Jiao

**Affiliations:** ^1^ HPB Surgical Unit, Department of Surgery and Cancer, Imperial College, London, UK; ^2^ Division of Cancer, Department of Surgery and Cancer, Imperial College, London, UK; ^3^ Department of Medical Oncology, VU University Medical Center, Cancer Center Amsterdam, Amsterdam, The Netherlands; ^4^ Cancer Pharmacology Lab, AIRC Start-Up Unit, University of Pisa, Pisa, Italy; ^5^ University of Sussex, School of Life Sciences, John Maynard Smith Building, Falmer, Brighton, UK

**Keywords:** Glypican-1 (GPC1), pancreatic ductal adenocarcinoma (PDAC), exosome, biomarker, tumor size

## Abstract

**Background:**

Glypican-1 (GPC1) is expressed in pancreatic ductal adenocarcinoma (PDAC) cells and adjacent stromal fibroblasts. Recently, GPC1 circulating exosomes (crExos) have been shown to be able to detect early stages of PDAC. In this study, we investigated the usefulness of crExos GPC1 as a biomarker for PDAC.

**Methods:**

Plasma was obtained from patients with benign pancreatic disease (*n* = 16) and PDAC (*n* = 27) prior to pancreatectomy, and crExos were isolated by ultra-centrifugation. Protein was extracted from surgical specimens (adjacent normal pancreas, *n* = 13; and PDAC, *n* = 17). GPC1 levels were measured using enzyme-linked immunosorbent assay (ELISA).

**Results:**

There was no significant difference in GPC1 levels between normal pancreas and PDAC tissues. This was also true when comparing matched pairs. However, GPC1 levels were enriched in PDAC crExos (*n* = 11), compared to the source tumors (*n* = 11; 97 ± 54 vs. 20.9 ± 12.3 pg/mL; *P* < 0.001). In addition, PDACs with high GPC1 expression tended to have crExos with higher GPC1 levels. Despite these findings, we were unable to distinguish PDAC from benign pancreatic disease using crExos GPC1 levels. Interestingly, we found that in matched pre and post-operative plasma samples there was a significant drop in crExos GPC1 levels after surgical resection for PDAC (*n* = 11 vs. 11; 97 ± 54 vs. 77.8 ± 32.4 pg/mL; *P* = 0.0428). Furthermore, we found that patients with high crExos GPC1 levels have significantly larger PDACs (>4 cm; *P* = 0.012).

**Conclusions:**

High GPC1 crExos may be able to determine PDAC tumor size and disease burden. However, further efforts are needed to elucidate its role as a diagnostic and/or prognostic biomarker using larger cohorts of PDAC patients.

## INTRODUCTION

Pancreatic ductal adenocarcinoma (PDAC) is a lethal disease and diagnostic difficulties result in late presentation and poor survival outcomes. Glypicans are a family member of heparan sulfate proteoglycans (HSPGs). HSPGs are diverse, ubiquitous and highly abundant macromolecules located on cell-surfaces, extracellular matrices and connective tissues [1]. They have been involved in broad cellular processes, including cell recognition, growth and adhesion, as well as proliferation, differentiation and morphogenesis [1]. Moreover, defective expression of HSPGs has been observed in human malignancies [2–4].

Earlier studies have shown that GPC1 is over-expressed in human PDAC cells and their adjacent fibroblasts [4], as well as in a significant percentage of gliomas [3], breast [2] and ovarian tumors [5]. Furthermore, silencing of GPC1 in breast cancer [2] and PDAC cells [6] leads to a decrease in tumor growth and an attenuation of mitogenic responses. Indeed, down-regulation of GPC1 in PDAC cells is associated with reduced proliferation and a reduction in tumor growth, angiogenesis and metastasis *in vitro* and *in vivo* [7].

Exosomes are small vesicles (50–140 nm in size) of endocytic origin that contain specific proteins, lipids and nucleic acids characteristic of their cellular origin [8]. They are secreted by different types of cells, including cancer cells, and they have been reported to be essential in cell-cell communication between cancer cells and their environment transferring information via their cargo [8]. Their main functions are to promote cancer development, stimulate angiogenesis, activate fibroblasts within the stroma, generate a pre-metastatic niche and inhibit host immune responses [9–11].

Recently, Melo *et al.* isolated circulating exosomes positive for glypican-1 (crExos GPC1^+^) from the serum of patients with PDAC and found that GPC1^+^ crExos could detect all stages of disease with unrivalled sensitivity and specificity [12]. Indeed, GPC1 was highly expressed on crExos in early and late stages of PDAC, compared to healthy controls (HC) and those with benign pancreatic disease [12].

In this study, we used ELISA to quantify GPC1 levels in pancreatic tissues and crExos.

## RESULTS

**Figure 1:**
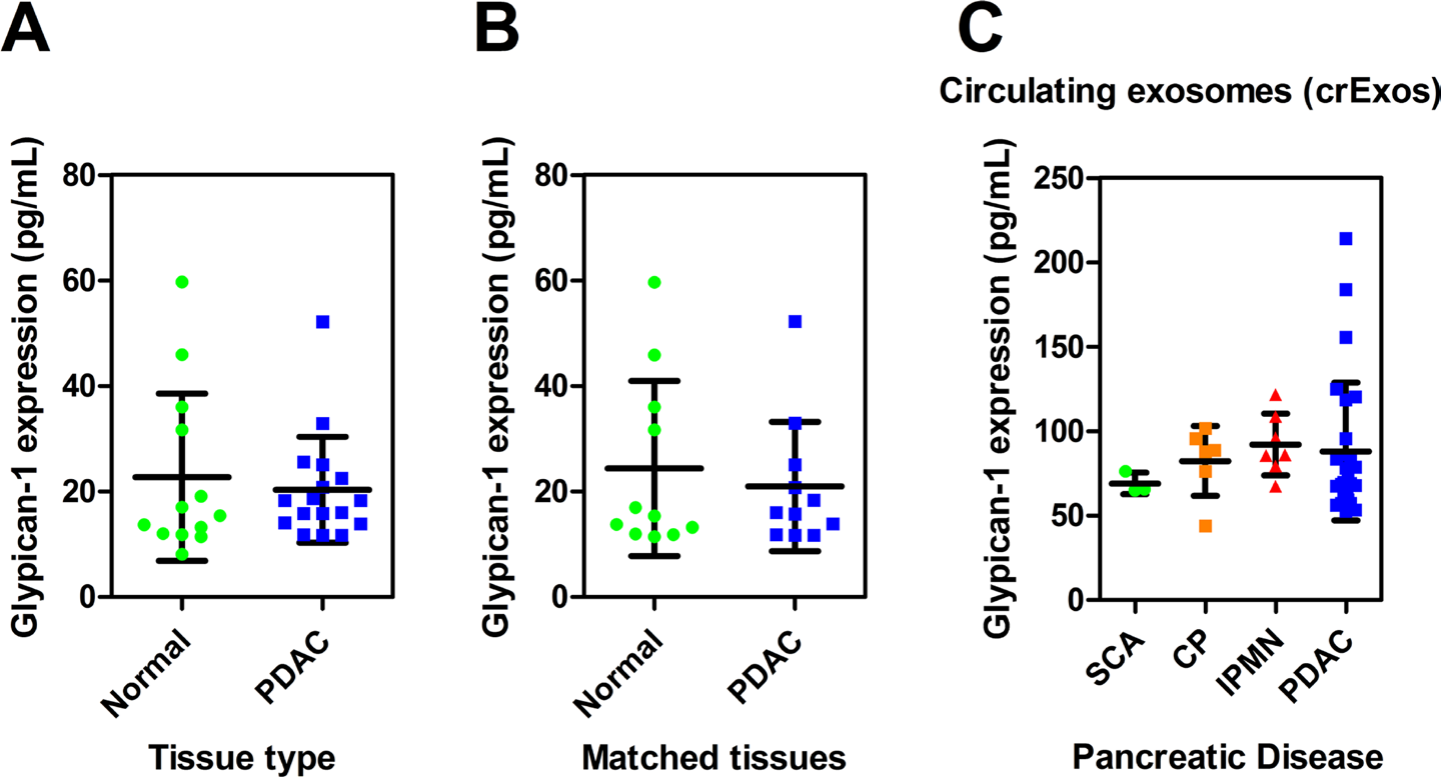
Glypican-1 (GPC1) expression in pancreatic tissues and circulating exosomes (crExos) Displayed are the GPC1 protein levels measured by ELISA for (**A**) Adjacent normal pancreas (NP; *n* = 13) vs. PDAC (*n* = 17) tissues and (**B**) a sub-analysis for matched patient samples (*n* = 11 vs. 11). (**C**) GPC1 was measured in crExos from patients with benign pancreatic disease (BPD; total *n* = 16: intraductal papillary mucinous neoplasms (IPMN), *n* = 7; chronic pancreatitis (CP), *n* = 6; serous cystadenoma, SCA, *n* = 3); and PDAC (*n* = 27). There was no significant difference between BPD vs. PDAC, or between disease groups using a one-way analysis of variance (ANOVA) and Tukey’s post-hoc honest significant difference (HSD) test. Scatterplots show expression for each sample and the horizontal lines represent the mean expression level and standard deviation.

We isolated protein from PDAC tissues (*n* = 17) and adjacent normal pancreas (*n* = 13) taken at the time of pancreatectomy, and quantified GPC1 levels using ELISA (Figure 1A). Our results showed that there was no significant difference in GPC1 levels between normal pancreas and PDAC tissues (Figure 1A), and this was also true when comparing matched tissue pairs (*n* = 11 vs. 11; Figure 1B).

Next, pre-operative plasma crExos GPC1 levels were measured from patients undergoing surgery for benign pancreatic disease (*n* = 16) and PDAC (*n* = 27). Our benign pancreatic disease group contained 7 patients with intraductal papillary mucinous neoplasms (IPMN; 4 low/moderate grade dysplasia and 3 high grade dysplasia), 6 with chronic pancreatitis (CP), and 3 with serous cystadenomas (SCA). However, contrary to previous reports [12], we did not observe any significant difference in plasma crExos GPC1 levels between benign and malignant disease (Figure 1C). Although this might be due to a Type II error, it certainly indicates that crExos GPC1 may not be the best blood-based biomarker for diagnosing PDAC. Our assay achieved an area under the curve (AUC) of only 0.59, with a sensitivity of 74%, and a specificity of 44% for detecting PDAC.

**Figure 2:**
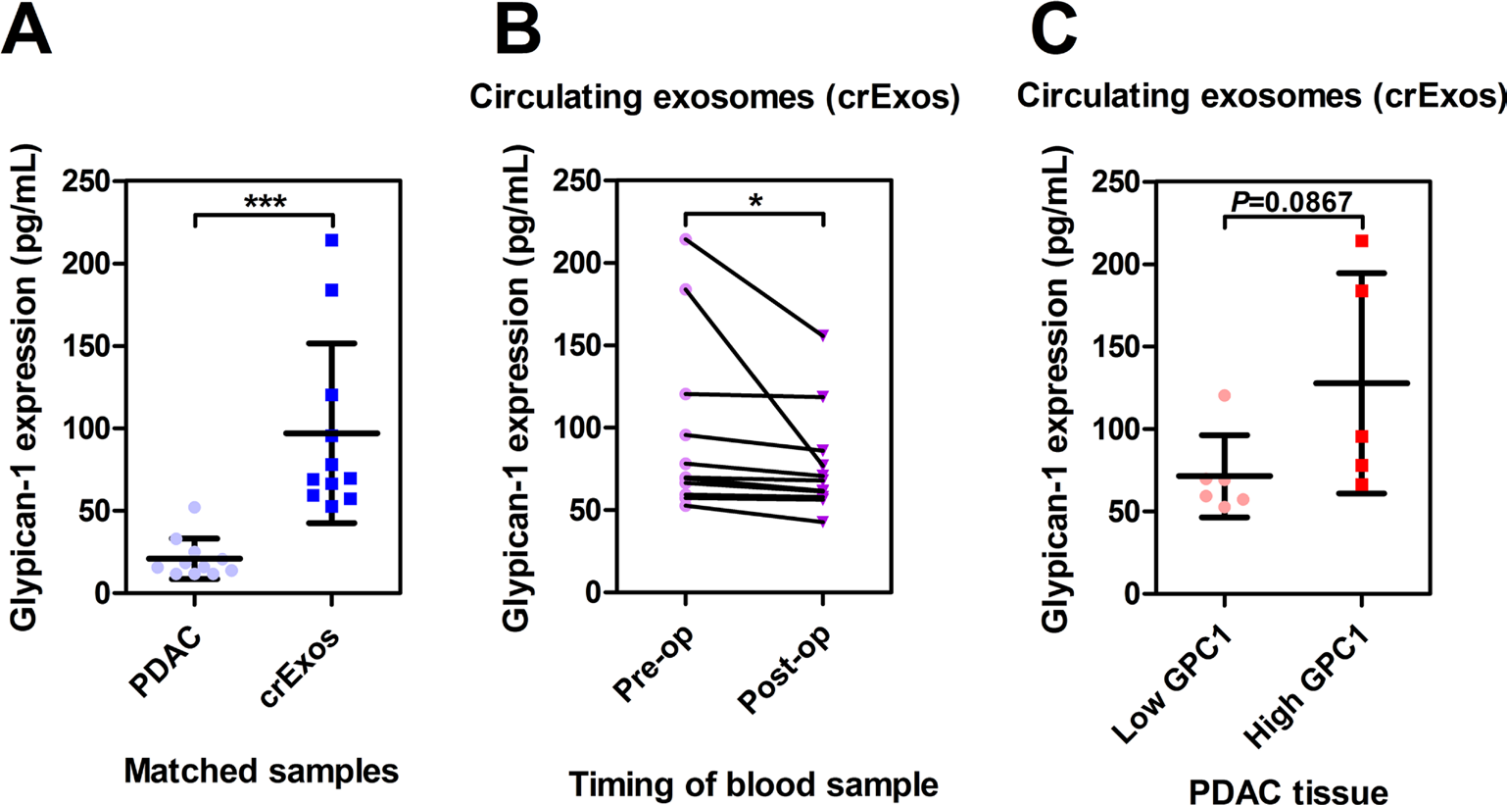
Circulating exosomal Glypican-1 (GPC1) is a marker of PDAC disease burden (**A**) In matched samples, Glypican-1 (GPC1) is enriched in circulating exosomes (crExos; *n* = 11) compared to their source PDAC tissues (*n* = 11; ^***^P < 0.001). (**B**) The crExos GPC1 levels were found to decrease post-operatively after surgical resection when compared to matched pre-operative samples from the same patients (*n* = 11 vs. 11; ^*^P < 0.05). (**C**) PDAC tissues with high GPC1 expression did not have crExos with higher GPC1 levels in matched samples.

Following on from this, we investigated our matched samples (i.e. plasma and tissues from the same patient), and found that GPC1 protein levels were higher in PDAC crExos (*n* = 11) in comparison to the originating tumoral tissues (*n* = 11; 97 ± 54 vs. 20.9 ± 12.3 pg/mL; *P* < 0.001; Figure 2A). This confirmed that GPC1 is enriched in PDAC crExos, as previously reported [12].

We then measured crExos GPC1 levels in matched pre and post-operative plasma samples (*n* = 11 vs. 11). These post-operative samples were taken at a mean of 48.7 days (range 28–82 days) after pancreatic resection, prior to any adjuvant therapy. We found that in these patients there was a drop in crExos GPC1 after surgical resection (97 ± 54 vs. 77.8 ± 32.4 pg/mL; *P* = 0.0428; Figure 2B). Although, we found no significant difference in post-operative crExos GPC1 levels based on resection margin status: R0 (*n* = 7) 76.2 ± 36.6 vs. R1 (*n* = 4) 80.7 ± 28.4 pg/mL; *P* > 0.05 (Supplementary Figure 1). This suggests that crExos GPC1 could be useful at monitoring gross tumor burden, but not microscopic residual disease.

Next, we hypothesized that patients with high crExos GPC1 pre-operatively may originate from PDACs with high GPC1 levels. We dichotomized tissue GPC1 levels into low or high expression in our 17 PDAC patients. We then assigned our 11 patients with matched crExos samples, according to whether these patients had high or low GPC1 expressing PDACs. However, we were unable to show a statistical difference in crExos GPC1 levels when stratifying by tumoral expression, although the numbers of patients in this analysis were small (Figure 2C).

Finally, we correlated both PDAC tissue GPC1 levels (*n* = 17) and pre-operative crExos GPC1 expression (*n* = 27) with clinico-pathological factors. This showed that tissue GPC1 levels were not significantly associated with any clinico-pathological factor (data not shown). However, high pre-operative crExos GPC1 expression levels were associated with larger tumor sizes (>4 cm; *P* = 0.012; Table 1).

**Table 1:** Summary of the clinico-pathological characteristics of the PDAC crExos cohort

Variables	Subcategory	PDAC crExos cohort *n* (%)	PDAC crExos Low GPC1 *n* (%)	PDAC crExos High GPC1 *n* (%)	*P* value^a^
**All cases^b^**		**27**	**13**	**14**	−
**Age, y**	≤60	6 (22.2)	3 (50)	3 (50)	0.918
	>60	21 (77.8)	10 (47.6)	11 (52.4)	
**Sex**	Female	11 (40.7)	6 (54.5)	5 (45.5)	0.581
	Male	16 (59.3)	7 (43.8)	9 (56.2)	
**Differentiation** **grade**	Low (G1/2) High (G3)	12 (44.4) 15 (55.6)	6 (50) 7 (46.7)	6 (50) 8 (53.3)	0.863
**Lymph-node** **Status (N)**	Absent (N0) Present (N1)	9 (33.3) 18 (66.7)	5 (55.6) 8 (44.4)	4 (44.4) 10 (55.6)	0.586
**Tumor Size, *cm***	<4	14 (51.9)	10 (71.4)	4 (28.6)	0.012
	>4	13 (48.1)	3 (23.1)	10 (76.9)	
**Perineural** **Invasion (PNI)**	No	8 (29.6)	4 (50)	4 (50)	0.901
	Yes	19 (70.4)	9 (47.4)	10 (52.6)	
**Resection margin** **Status (R)**	Negative (R0)	17 (63)	9 (52.9)	8 (47.1)	0.516
	Positive (R1)	10 (37)	4 (40)	6 (60)	

PDAC, pancreatic ductal adenocarcinoma; AJCC, American Joint Committee on Cancer; crExos, circulating exosomes; GPC1, Glypican-1. ^a^Pearson Chi-Square test; ^b^These samples were all pre-operative plasma crExos.

## DISCUSSION

In this study, we found that crExos GPC1 levels were not elevated in PDAC compared to benign pancreatic disease, when measured by ELISA. However, crExos GPC1 levels may be clinically useful for determining PDAC tumor size and disease burden. Previous studies have demonstrated an over-expression of GPC1 in PDAC tissues at the mRNA and protein levels [4, 13, 14]. The recent report by Melo *et al.* identified crExos GPC1^+^ as a highly accurate biomarker for the early detection of PDAC [12]. However, the techniques that they employed would be quite difficult to reproduce in a standard hospital laboratory, including the use of anti-GPC1 antibody labelled beads to bind crExos GPC1^+^, and subsequent quantification by flow cytometry. Therefore, we used ELISA to establish a more clinically amenable assay for the quantitative analysis of GPC1 levels in plasma crExos, whilst still being highly sensitive and reproducible.

We first examined GPC1 levels in adjacent normal pancreas and PDAC tissues. However, we were unable to differentiate between tissue types using GPC1 levels measured by ELISA. This may be due to the use of macro-dissected bulk tissues for protein extraction and GPC1 quantification. We may have had a more accurate analysis using laser micro-dissected tissues, since it is possible the adjacent normal pancreas could have had areas of desmoplastic stroma that were not easily visible during macro-dissection, and could have influenced the result. Alternatively, our ELISA assay may have detected GPC1 that had “leaked over” from the PDAC side of the transection margin. Kleeff *et al.* showed that there is an abundance of GPC1 in the fibroblasts surrounding PDAC and suggested that these cells participate in the storage of this growth factor [4]. As PDAC cells invade the surrounding stroma, this allows release of GPC1, leading to mitotic stimulation and tumor progression [4]. Indeed, Lu *et al.* found that in the TCGA analysis, which used bulk tissues, that GPC1 mRNA levels were higher in those patients with PDAC on a background of chronic pancreatitis (CP) [14]. They speculated that perhaps this inflammation increased GPC1 levels in these tissues, and also that there may have been some contamination from the desmoplastic stroma, which could have elevated GPC1 levels even further [14]. Immunohistochemistry (IHC), whilst being semi-quantitative, is able to visualise cellular localization of protein expression, and could be considered more accurate for determining differential expression between tissues that are not micro-dissected. Lu *et al.* determined distribution of GPC1 protein levels by IHC in normal pancreata (*n* = 2), CP (*n* = 4), adjacent normal pancreas (n = 169), and PDAC (n = 186) [14]. In this analysis, they found that 59.7% (111/186) of the PDAC tumors had positive cytoplasmic and membrane immunostaining for GPC1, whilst non-neoplastic tissues barely showed any GPC1 expression (2/175) [14]. In the positively stained PDAC tissues, 51.4% (57/111) had weak, 35.1% (39/111) had moderate, and 13.5% (15/111) had strong staining of GPC1 [14]. The other 40.3% (75/186) of PDACs had no immunostaining for GPC1 [14]. Similarly, Duan *et al.* found that whilst GPC1 expression was significantly higher in PDAC vs. normal pancreas, 43.5% (27/62) of PDACs were still negative for GPC1 [13]. Therefore, only ∼60% of PDACs are positive for GPC1 and those with GPC1 over-expression appear to have a more aggressive phenotype. Indeed, high GPC1 expression in PDAC tissues has been associated with perineural invasion (PNI), high grade, large tumor size and poor overall survival (OS) after surgical resection [13, 14]. This is unsurprising as GPC1 has been shown to enhance proliferation and migration *in vitro* [4], and promote tumor growth, angiogenesis, and invasion in mouse models of PDAC [15]. Based on these data, we correlated tissue GPC1 levels in our PDACs with clinico-pathological factors. However, we were unable to find any significant association, probably due to the small number of patients included.

Next, we sought to further investigate the ability of crExos GPC1 to diagnose PDAC. Several authors have asked for the results from Melo *et al.* [12] to be confirmed in an independent series of patients [16, 17]. These additional data would help to validate crExos GPC1 as a blood-based biomarker for the detection of PDAC [18]. Herreros-Villanueva *et al.* also suggested that the methodology should be optimized in order to achieve an accurate and less expensive biomarker, which is available in standard clinical laboratories, such as a simple blood test, without the need for exhaustive ultra-centrifugation steps and/or complicated staining techniques [18]. We have made the first step towards this by measuring crExos GPC1 levels using ELISA, which was a sensitive, simple and reproducible technique. However, we still employed recognised ultra-centrifugation techniques to isolate plasma crExos [19]. Using these methods, we were able to confirm that crExos are enriched in GPC1 compared to their originating PDAC tissues. The presence of crExos GPC1^+^ has been reported to be near perfect for distinguishing PDAC from benign pancreatic disease and healthy controls, with an AUC of 1.0, and 100% sensitivity and specificity [12]. However, when we measured pre-operative crExos GPC1 levels by ELISA in patients undergoing pancreatectomy for benign pancreatic disease and PDAC, we were unable to discriminate between the two groups with any statistical significance, and found a disappointing AUC of 0.59. This may have been due to small sample sizes, or that ELISA was too insensitive to accurately quantify crExos GPC1 levels. Interestingly, when Melo *et al.* [12] used an ELISA assay to detect circulating free GPC1 in the serum of healthy controls, and patients with benign pancreatic disease and PDAC, this exhibited lower sensitivity and specificity (82% and 75% respectively; AUC 0.78) for detecting PDAC compared to measuring crExos GPC1^+^ by flow cytometry. Our study has been the first to use ELISA to quantify crExos GPC1 levels. Remarkably, Lai *et al.* also found considerable overlap in pre-operative plasma crExos GPC1 levels between healthy controls, CP and PDAC patients when measured by liquid chromatography-tandem mass spectrometry (LC-MS/MS), resulting in an AUC of 0.75 for detecting PDAC [20]. Thus, crExos GPC1 may not have the high sensitivity and specificity for detecting PDAC as previously reported [12], but this could be dependent on the methods used. Melo *et al.* found that crExos GPC1^+^ levels were consistently higher in patients with intraductal papillary mucinous neoplasms (IPMN), compared to healthy controls, or patients with CP or SCA [12]. Our benign pancreatic disease group contained 7 patients with IPMN, 6 with CP, and 3 with SCA. However, we did not find a significant difference in crExos GPC1 levels between these groups after using a multiple comparison test.

Tumoral GPC1 levels have been correlated with worse biological characteristics in PDAC. We hypothesized that crExos GPC1 levels may also be associated with clinico-pathological features, and found that high crExos GPC1 expression was associated with larger tumor sizes (>4 cm). Indeed, Lu *et al.* previously observed that higher tumoral GPC1 protein levels were found in very large PDACs (>6 cm) [14]. Interestingly, when we examined matched tumor and plasma samples, we found that PDACs with high GPC1 protein levels tended to produce crExos with higher GPC1 levels (127.7 ± 66.8 vs. 71.5 ± 24.9 pg/mL), but this was not statistically significant. Looking at the longitudinal plasma samples collected pre- and post-operatively from these patients, we found that ∼7 weeks after pancreatic resection there was a significant reduction in crExos GPC1 levels. However, there was no significant difference in post-operative crExos GPC1 levels when comparing R0 vs. R1 resections. These data indicate that crExos GPC1 may be a useful biomarker for estimating gross PDAC tumor burden, and are in line with findings by Melo and colleagues [12]. Furthermore, Melo *et al.* found a significant decrease in crExos GPC1^+^ levels at 7 days after surgical resection of a PDAC, or even an IPMN [12]. Lai *et al.* found that after resection of a PDAC there was a non-significant trend towards reduced crExos GPC1 levels post-operatively, but they admit this was probably because they only investigated 3 patients in this analysis [20]. Since crExos GPC1 levels are also elevated in patients with breast [12] and colorectal cancers [21], this suggests that it would not be a good biomarker for screening for PDAC, but rather to monitor known disease or perhaps be used as an adjunct investigation in patients with suspected PDAC.

Cancer exosomes are mediators of cell-to-cell communication and carry tumor-promoting cargo to recipient cells [10]. Proteins, lipids, DNA and RNAs (mRNAs, microRNAs and other non-coding RNAs) are present in tumor-derived exosomes and are crucial players in exosome biology. Therefore, exosomes have the potential to be clinically useful diagnostic and/or prognostic biomarkers in cancer. Indeed, this may also be cost effective, as the isolation and use of exosomal biomarkers from patients would certainly be cheaper, less invasive, and potentially more sensitive and specific than many of the current clinical diagnostic tests [22]. Melo and colleagues reported that crExos GPC1^+^ is an attractive candidate for identifying patients with early and more established PDAC [12], as well as potentially malignant pancreatic precursor lesions (i.e. mucinous cystic neoplasms) [23]. However, we agree with Diamandis and co-authors [17] that there are still no biomarkers that perform with 100% specificity and sensitivity for PDAC. Indeed, we have shown that crExos GPC1 measured by ELISA may be useful as a marker of tumor burden and response to surgical resection, but we were unable to distinguish benign pancreatic disease from PDAC pre-operatively. It is now essential to standardize methods for exosome isolation and crExos GPC1 quantification, in order to ultimately validate this biomarker as clinically relevant for patients with suspected pancreatic tumors.

## METHODS

### Ethics statement

This study was approved by a London Research Ethics Committee (Camden & Islington 09/H0722/77, 26th November 2009). All patients signed an informed consent form for research prior to a venepuncture being performed and tissue sampling.

### Study population

The clinico-pathological features of selected patients are summarized in Table 1. A total of 27 PDAC patients with stage T2-3, N0-1, M0 underwent pancreatic resection.

### Plasma and tissue samples

Pre-operative blood samples from 16 patients with benign pancreatic disease and 27 patients with PDAC undergoing pancreatic surgery were obtained at Hammersmith Hospital. Blood (5–10 ml) was collected into EDTA-coated tubes for all patients prior to surgery, and ∼7 weeks following surgery for PDAC for 11 patients. Specimens were either placed on ice or in a refrigerator (4° C), or taken directly to the lab and rapidly processed by centrifugation (1000 × g for 10 min) at 4° C (within 2 hours of collection). Plasma supernatants were collected and stored at −80° C until required. Tissue extracts from patients (PDAC, *n* = 17; and adjacent normal pancreas, *n* = 13) were obtained intra-operatively at Hammersmith Hospital and stored directly at −80° C until required. There were matched samples for 11 patients.

### Protein extraction from tissues

Tissue lysates were prepared from minced frozen samples of PDAC and adjacent normal pancreas on ice in a lysis buffer (50 mM Tris [pH 7.6], 0.5 M NaCl, 0.1% sodium dodecyl sulfate [SDS], 0.02% NaN_3_, 1 mM phenylmethanesulfonyl fluoride [PMSF], 5 mM ethylenediaminetetraacetic acid [EDTA], and 1 mM iodoacetamide) containing protease inhibitor cocktail (Roche, Basel, Switzerland), homogenized overnight at 4° C, and centrifuged at 10,000 g for 10 min at 4° C. Supernatants were kept frozen at −80° C until required. Protein concentrations of the lysates were determined using a Pierce BCA assay kit (Thermo Fisher Scientific, Rockford, USA).

### Isolation of exosomes and quantification of GPC1

Isolation of crExos from the plasma (1–1.5 ml) of patients with PDAC and BD was performed by ultra-centrifugation using the protocols by Théry *et al.* [19]. GPC1 levels in crExos and tissue lysates were quantified by enzyme-linked immunosorbent assay (ELISA; E9038h; 2BScientific Ltd, UK) according to the manufacturer’s protocol. The tetraspanins CD9, CD63, and CD81 are classically used as exosome markers [24]. We used the Exo-Check antibody array (System Biosciences, UK) to check exosomal marker expression following the manufacturer’s protocol. This array has 12 pre-printed spots and 8 antibodies for known exosome markers (CD63, CD81, ALIX, FLOT1, ICAM1, EpCAM, ANXA5 and TSG101), and a positive control spot derived from human serum exosome proteins. Supplementary Figure 2 shows representative blots confirming exosomal markers in our plasma crExos samples, including CD63 and/or CD81 expression.

### Statistical analyses

All experimental data are reported as means; error bars represent standard deviation (SD). Comparisons between groups were performed using paired or unpaired, 1 or 2-tailed Student’s *t*-tests where appropriate. Correlations between GPC1 expression and clinico-pathological characteristics were calculated using χ^2^ test. To compare multiple groups, a one-way analysis of variance (ANOVA) and Tukey’s post-hoc honest significant difference (HSD) test was used. Statistical analyses were performed using GraphPad Prism 7.0 or SPSS for Windows version 20.0 (IBM SPSS Statistics, Chicago, IL, USA). *P*-values < 0.05 were considered significant.

## SUPPLEMENTARY MATERIALS FIGURES



## References

[1] Perrimon N, Bernfield M (2000). Specificities of heparan sulphate proteoglycans in developmental processes. Nature.

[2] Matsuda K, Maruyama H, Guo F, Kleeff J, Itakura J, Matsumoto Y, Lander AD, Korc M (2001). Glypican-1 is overexpressed in human breast cancer and modulates the mitogenic effects of multiple heparin-binding growth factors in breast cancer cells. Cancer Res.

[3] Su G, Meyer K, Nandini CD, Qiao D, Salamat S, Friedl A (2006). Glypican-1 is frequently overexpressed in human gliomas and enhances FGF-2 signaling in glioma cells. Am J Pathol.

[4] Kleeff J, Ishiwata T, Kumbasar A, Friess H, Büchler MW, Lander AD, Korc M (1998). The cell-surface heparan sulfate proteoglycan glypican-1 regulates growth factor action in pancreatic carcinoma cells and is overexpressed in human pancreatic cancer. The Journal of clinical investigation.

[5] Davies EJ, Blackhall FH, Shanks JH, David G, McGown AT, Swindell R, Slade RJ, Martin-Hirsch P, Gallagher JT, Jayson GC (2004). Distribution and clinical significance of heparan sulfate proteoglycans in ovarian cancer. Clinical cancer research.

[6] Li J, Kleeff J, Kayed H, Felix K, Penzel R, Buchler MW, Korc M, Friess H (2004). Glypican-1 antisense transfection modulates TGF-beta-dependent signaling in Colo-357 pancreatic cancer cells. Biochem Biophys Res Commun.

[7] Aikawa T, Whipple CA, Lopez ME, Gunn J, Young A, Lander AD, Korc M (2008). Glypican-1 modulates the angiogenic and metastatic potential of human and mouse cancer cells. J Clin Invest.

[8] Thery C (2011). Exosomes: secreted vesicles and intercellular communications. F1000 Biol Rep.

[9] Costa-Silva B, Aiello NM, Ocean AJ, Singh S, Zhang H, Thakur BK, Becker A, Hoshino A, Mark MT, Molina H, Xiang J, Zhang T, Theilen TM (2015). Pancreatic cancer exosomes initiate pre-metastatic niche formation in the liver. Nature cell biology.

[10] Bastos N, Ruivo CF, da Silva S, Melo SA (2017 Aug 11). Exosomes in cancer: Use them or target them? Seminars in Cell & Developmental Biology.

[11] Whiteside TL (2016). Exosomes and tumor-mediated immune suppression. Journal of Clinical Investigation.

[12] Melo SA, Luecke LB, Kahlert C, Fernandez AF, Gammon ST, Kaye J, LeBleu VS, Mittendorf EA, Weitz J, Rahbari N, Reissfelder C, Pilarsky C, Fraga MF (2015). Glypican-1 identifies cancer exosomes and detects early pancreatic cancer. Nature.

[13] Duan L, Hu XQ, Feng DY, Lei SY, Hu GH (2013). GPC-1 may serve as a predictor of perineural invasion and a prognosticator of survival in pancreatic cancer. Asian Journal of Surgery.

[14] Lu H, Niu F, Liu F, Gao J, Sun Y, Zhao X (2017). Elevated glypican-1 expression is associated with an unfavorable prognosis in pancreatic ductal adenocarcinoma. Cancer Med.

[15] Whipple CA, Young AL, Korc M (2012). A KrasG12D-driven genetic mouse model of pancreatic cancer requires glypican-1 for efficient proliferation and angiogenesis. Oncogene.

[16] Lorenzon L, Blandino G (2016). Glypican-1 exosomes: do they initiate a new era for early pancreatic cancer diagnosis?. Translational Gastroenterology and Hepatology.

[17] Diamandis EP, Plebani M (2016). Glypican-1 as a highly sensitive and specific pancreatic cancer biomarker. Clin Chem Lab Med.

[18] Herreros-Villanueva M, Bujanda L (2016). Glypican-1 in exosomes as biomarker for early detection of pancreatic cancer. Annals of translational medicine.

[19] Thery C, Amigorena S, Raposo G, Clayton A (2006). Isolation and characterization of exosomes from cell culture supernatants and biological fluids. Current protocols in cell biology.

[20] Lai X, Wang M, McElyea SD, Sherman S, House M, Korc M (2017). A microRNA signature in circulating exosomes is superior to exosomal glypican-1 levels for diagnosing pancreatic cancer. Cancer Letters.

[21] Li J, Chen Y, Guo X, Zhou L, Jia Z, Peng Z, Tang Y, Liu W, Zhu B, Wang L, Ren C (2017). GPC1 exosome and its regulatory miRNAs are specific markers for the detection and target therapy of colorectal cancer. Journal of cellular and molecular medicine.

[22] Nuzhat Z, Kinhal V, Sharma S, Rice GE, Joshi V, Salomon C (2017). Tumour-derived exosomes as a signature of pancreatic cancer - liquid biopsies as indicators of tumour progression. Oncotarget.

[23] Moutinho-Ribeiro P, Silva S, Adem B, Silva M, Lopes S, Vilas-Boas F, Melo S, Macedo G (2017). Glipican-1 circulating exosomes levels are higher in pancreatic adenocarcinoma and cystic mucinous neoplasms than in other associated risk groups for pancreatic cancer: Clues for early diagnosis?. Pancreatology.

[24] Kowal J, Arras G, Colombo M, Jouve M, Morath JP, Primdal-Bengtson B, Dingli F, Loew D, Tkach M, Théry C (2016). Proteomic comparison defines novel markers to characterize heterogeneous populations of extracellular vesicle subtypes. Proceedings of the National Academy of Sciences.

