# Supplementary material for: Glypican-1 is enriched in circulating-exosomes in pancreatic cancer and correlates with tumor burden
Source: Oncotarget. 2018 Apr 10;9(27):19006–13. doi: 10.18632/oncotarget.24873 (PMC5922373; doi:10.18632/oncotarget.24873)
Supplement: Supplementary file 1 [file oncotarget-09-19006-s001.pdf]

Glypican-1 is enriched in circulating-exosomes in pancreatic cancer and correlates with tumor burden

SUPPLEMENTARY MATERIALS

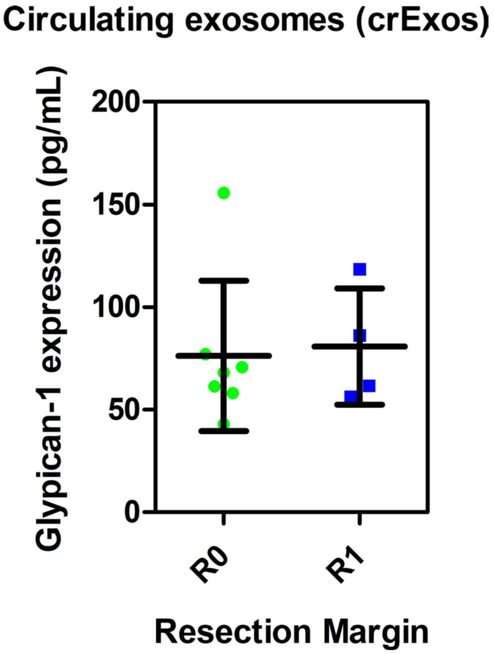

Supplementary Figure 1: Post-operative circulating exosomal Glypican-1 levels are not associated with resection margin status. There was no difference in post-operative crExos Glypican-1 levels when comparing R0 (*n* = 7) vs. R1 (*n* = 4) resections.

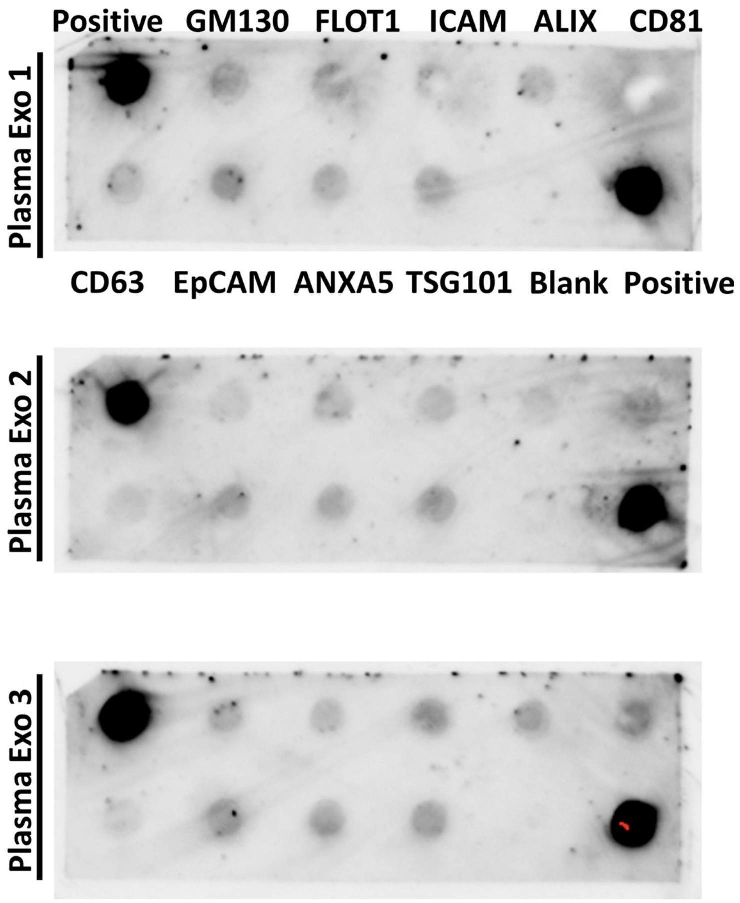

Supplementary Figure 2: Exo-Check antibody array showing expression of exosomal markers in plasma crExos from patients. The arrays confirmed expression of exosomal markers in our patient plasma crExos samples, including CD63 and/or CD81. Three representative blots are shown.
